# Possible role of lymphocytes in glucocorticoid-induced increase in trabecular bone mineral density

**DOI:** 10.1530/JOE-14-0508

**Published:** 2015-01

**Authors:** Louise Grahnemo, Caroline Jochems, Annica Andersson, Cecilia Engdahl, Claes Ohlsson, Ulrika Islander, Hans Carlsten

**Affiliations:** 1 Department of Rheumatology and Inflammation Research, Centre for Bone and Arthritis Research, Institute of Medicine, The Sahlgrenska Academy, University of Gothenburg, Box 480, Gothenburg, 405 30, Sweden; 2 Department of Internal Medicine and Clinical Nutrition, Centre for Bone and Arthritis Research, Institute of Medicine, The Sahlgrenska Academy, University of Gothenburg, Box 480, Gothenburg, 405 30, Sweden; 3 Laboratory of Tumor Immunology and Biology, Center for Cancer Research, National Cancer Institute, National Institutes of Health, Bethesda, Maryland, USA

**Keywords:** glucocorticoid, osteoporosis, immune system, estrogen

## Abstract

Treatment with anti-inflammatory glucocorticoids is associated with osteoporosis. Many of the treated patients are postmenopausal women, who even without treatment have an increased risk of osteoporosis. Lymphocytes have been shown to play a role in postmenopausal and arthritis-induced osteoporosis, and they are targeted by glucocorticoids. The aim of this study was to investigate the mechanisms behind effects of glucocorticoids on bone during health and menopause, focusing on lymphocytes. Female C57BL/6 or SCID mice were therefore sham-operated or ovariectomized and 2 weeks later treatment with dexamethasone (dex), the nonsteroidal anti-inflammatory drug carprofen, or vehicle was started and continued for 2.5 weeks. At the termination of experiments, femurs were phenotyped using peripheral quantitative computed tomography and high-resolution micro-computed tomography, and markers of bone turnover were analyzed in serum. T and B lymphocyte populations in bone marrow and spleen were analyzed by flow cytometry. Dex-treated C57BL/6 mice had increased trabecular bone mineral density, but lower cortical content and thickness compared with vehicle-treated mice. The dex-treated mice also had lower levels of bone turnover markers and markedly decreased numbers of spleen T and B lymphocytes. In contrast, these effects could not be repeated when mice were treated with the nonsteroidal anti-inflammatory drug carprofen. In addition, dex did not increase trabecular bone in ovariectomized SCID mice lacking functional T and B lymphocytes. In contrast to most literature, the results from this study indicate that treatment with dex increased trabecular bone density, which may indicate that this effect is associated with corticosteroid-induced alterations of the lymphocyte populations.

## Introduction

Glucocorticoids are frequently used for the treatment of noninfectious and autoimmune inflammation as, amongst other mechanisms, they can suppress lymphocytes (Chantler *et al*. 2003, Busillo & Cidlowski 2013). Prolonged use of glucocorticoids is associated with several adverse side effects such as bone loss (Silverman & Lane 2009). Although the results of most studies indicate that glucocorticoids have detrimental effects on bone, data are conflicting due to underlying inflammatory disease, dose, and duration of treatment. Patients with early rheumatoid arthritis, treated with a combination of prednisolone and disease-modifying anti-rheumatic drugs over a period of 2 years, had an increased remission rate and fewer eroded joints (Svensson *et al*. 2005). In addition, the degree of bone loss was similar to that in controls. Patients treated with low doses of glucocorticoids for insufficient levels of endogenous glucocorticoids, caused by primary adrenal insufficiency or congenital adrenal hyperplasia, showed bone mineral densities within the normal range (Koetz *et al*. 2012). Similar results have also been obtained using an experimental model of postmenopausal arthritis where the trabecular bone mineral density (BMD) in dexamethasone (dex)-treated ovariectomized arthritic mice was similar to that in controls (Islander *et al*. 2011*a*). Based on these and other studies (Binz *et al*. 1994, King *et al*. 1996, Kugelberg *et al*. 2005, Ogoshi *et al*. 2008, Kwok *et al*. 2012), there are still questions regarding the effects of glucocorticoids on bone.

As in the experimental model of postmenopausal arthritis, many of the patients treated with glucocorticoids are postmenopausal women (Van Staa *et al*. 2000*a*). Even without glucocorticoid treatment, these women have a high risk of developing osteoporosis; approximately 20% of women in Europe over 50 years of age are affected by postmenopausal osteoporosis. The lifetime fracture risk in these women is over 45% (Hernlund *et al*. 2013). The primary reason for this is loss of estrogen, which results in an increased bone turnover (Garnero *et al*. 1996, Riggs *et al*. 1998), most probably induced by a subclinical inflammation (Mundy 2007). Activated immune cells produce pro-inflammatory cytokines, which inhibit osteoblast maturation, induce osteoblast apoptosis, and increase osteoclast activity and maturation, thus favoring bone resorption (Kurihara *et al*. 1990, Jilka *et al*. 1992, Jimi *et al*. 1999, Kotake *et al*. 1999, Tsuboi *et al*. 1999, Gilbert *et al*. 2000, Kobayashi *et al*. 2000). The sources of these cytokines are believed to be monocytes/macrophages, stromal cells/osteoblasts, and T lymphocytes (Riggs *et al*. 2002). In particular, the T lymphocytes have been suggested to be a major source of tumor necrosis factor during estrogen deficiency (Pacifici 2008).

T and B lymphocytes are well-known targets of glucocorticoids (Fauci 1976, Tuckermann *et al*. 2005). The aim of this study was to investigate the mechanisms behind the effects of glucocorticoids on bone during health and menopause, focusing on lymphocytes. We used ovariectomy (ovx) operated mice as a model for postmenopausal osteoporosis. Ovx and sham-operated female mice were treated with the glucocorticoid dex and bone morphology was analyzed. We also investigated the bone morphology in ovariectomized dex-treated SCID mice lacking functional T and B lymphocytes.

## Materials and methods

### Animals

This study was approved by the ethical committee for animal experiments in Gothenburg (Permit numbers: 72-2004, 49-2007, and 160-2012) and all efforts were made to minimize suffering. Female C57BL/6 mice (Scanbur AB, Sollentuna, Sweden) and SCID mice (Taconic M&B A/S, Ry, Denmark) were electronically tagged and kept, five to ten animals per cage, under standard environmental conditions, had access to standard laboratory chow and tap water and were allowed to eat and drink *ad libitum*.

### Castration

C57BL/6 and SCID mice were either sham-operated or ovariectomized at 8–10 weeks of age. Ovaries were removed through a midline incision of the skin, and flank incisions of the peritoneum. The skin incision was then closed with metallic clips. Sham-operated mice had their ovaries exposed, but not removed. Surgery was performed after the mice were anesthetized with isoflurane (Baxter Medical AB, Kista, Sweden) or ketamine (Pfizer AB) and medetomidine (Orion Pharma, Espoo, Finland). Carprofen (carp; Orion Pharma) was used for post-operative analgesia.

### Treatment

The mice received i.p. injections of 5 days/week (days 1–5, 8–12, and 15–16) of the synthetic corticosteroid dex (Oradexon, Organon, Gothenburg, Sweden or Dexadreson vet, Intervet, Sollentuna, Sweden) (1, 12.5, 50, 125, or 250 μg/mouse) or s.c. injections 7 days/week of the nonsteroidal anti-inflammatory drug (NSAID) carp (Orion Pharma) (125 μg/mouse) dissolved in 0.9% (w/v) sodium chloride (in total 100 μl). The dose used for carp-treatment was chosen based on the standard post-operative dose used in mice (Flecknell & Waterman-Pearson 2000, Raoul *et al*. 2005, Koch *et al*. 2010, Senchenkova *et al*. 2011), and in rats it has been shown to effectively inhibit inflammation (Essien & Kotiw 2012). Control mice received i.p. or s.c. injections of 0.9% (w/v) sodium chloride (100 μl/mouse per day). Treatment with dex, carp, or vehicle was started approximately 2 weeks after surgery and continued for 2.5 weeks (terminated day 17), as this duration of treatment is sufficient to produce bone effects with another steroid, estradiol.

### Tissue collection

At the end of the experiments, mice were anesthetized with ketamine and medetomidine before blood collection and then killed by cervical dislocation. Sera were individually collected and stored at −20 °C until use. Successful removal of the ovaries during the surgical procedure was confirmed by weighing the uteri. One femur was placed in 10% (v/v) phosphate-buffered formalin and then in 70% (v/v) ethanol before analysis of BMD. The other femur was used for flow cytometry of bone marrow cells. One tibia was dissected for biomechanical testing. Thymus and spleen weight were recorded.

### Peripheral quantitative computed tomography

One femur was subjected to a peripheral quantitative computed tomography (pQCT) scan with a Stratec pQCT XCT Research M, software version 5.4B (Norland, Fort Atkinson, WI, USA) at a resolution of 70 μm, as described previously (Windahl *et al*. 1999). Trabecular BMD was determined by a metaphyseal scan at a point 3% of the length of the femur from the growth plate. The inner 45% of the area was defined as the trabecular bone compartment. A mid-diaphyseal scan at a point 36% of the length of the femur from the growth plate was performed to determine cortical content, thickness, and BMD.

### High-resolution micro computed tomography (μCT)

High-resolution μCT analyses were performed on the distal femur by using an 1172 model μCT (Bruker micro-CT, Aartselaar, Belgium). The femurs were imaged with an X-ray tube voltage of 50 kV and current of 201 μA, with a 0.5-mm aluminium filter. The scanning angular rotation was 180° and the angular increment 0.70°. The voxel size was 4.48 μm isotropically. NRecon (version 1.6.9) was employed to perform the reconstruction following the scans. The trabecular bone proximal to the distal growth plate was selected for analyses within a conforming volume of interest (cortical bone excluded) commencing at a distance of 650 μm from the growth plate, and extending a further longitudinal distance of 134.5 μm in the proximal direction. Cortical measurements were performed in the diaphyseal region starting at a distance of 3.59 mm from the growth plate and extending a further longitudinal distance of 134.5 μm in the proximal direction.

### Serologic markers of bone remodeling

Bone resorption was assessed using serum levels of C-terminal telopeptides of type 1 collagen (CTX1) using ELISA (Nordic Bioscience Diagnostics, Herlev, Denmark) according to the manufacturer's instructions. Serum levels of osteocalcin, a marker of bone formation, were determined with a Mouse Osteocalcin IRMA kit (Immutopics, Inc., San Clemente, CA, USA). The detection limits for CTX1 and osteocalcin were 6 and 0.1 ng/ml respectively.

### Bone strength

Biomechanical properties of tibial shafts were studied using a three-point bending test. The test was performed using an Instron 3343 biomechanical testing system and M-Bluehill-K2-EN Software revision A (Instron, Norwood, MA, USA). Before biomechanical testing, tibiae were dissected free of surrounding tissues, wrapped in PBS-soaked paper, and stored at −20 °C. Just before testing, tibiae were thawed and the rest of the soft tissue was removed. After the temperature of the samples had reached room temperature, tibiae were placed in a stable position on supports, with a spacing of 6 mm and the tibia–fibula junction at the top. The cross head of the biomechanical testing system was placed so that the head was in contact with the bone surface. The test was started using a displacement interval of 3 mm/min. Applied load and displacement were recorded throughout the test using a data collection rate of 50 ms. The test was ended when the rate of flexure load had decreased by 15%. The following parameters were then determined using built-in algorithms provided by the biomechanical testing system: maximal load (N), energy absorption at maximal load (mJ) (describes the toughness of the sample), and modulus (MPa) (describes the stiffness of the sample).

### Flow cytometry analysis

One femur was flushed with PBS through the bone cavity to harvest bone marrow cells. The spleens were removed and single-cell suspensions were prepared by pressing the organs through 70 μm cell strainers (Becton Dickinson, Franklin Lakes, NJ, USA). A Tris-buffered 0.83% (w/v) NH_4_Cl solution, pH 7.29, was used to lyse erythrocytes, and the cells were washed and re-suspended in fluorescence-activated cell sorting (FACS)-buffer (PBS supplemented with 1% (v/v) FCS and 0.1% (v/v) NaAz). Labeling of cell surface markers was performed using anti-CD19 PerCP (BioLegend, San Diego, CA, USA), anti-CD3 APC (BioLegend), anti-CD4 V500 (Becton Dickinson), and anti-CD8 FITC antibodies (Becton Dickinson). Lymphocytes were gated on singlet cells and thereafter B cells were defined as CD19^+^ lymphocytes, mature CD4^+^ T cells as CD4^+^CD3^+^ lymphocytes, and mature CD8^+^ T cells as CD8^+^CD3^+^ lymphocytes. The samples were run on a Becton Dickinson FACS Canto II and data was analyzed using the Flow Jo 10.0.6 Software (Three Star, Inc, Ashland, OR, USA).

### Statistical analyses

Statistical analyses were performed using the SPSS Software (version 21.0.0.0 for Windows). For statistical evaluation, ANOVA with Tukey's *post hoc* test was used unless Levene's test revealed unequal variance, then Dunnett's T3 test was used. Logarithmic transformations were used when appropriate to ensure normal distribution of data. Dose dependence was analyzed with linear regression, and the presented *β* values are unstandardized. All tests are two-sided. Data are presented as arithmetical mean±s.e.m. or geometric mean±95% CI when logarithmic data are used, unless otherwise stated. *P*<0.05 was considered significant.

## Results

### Treatment with dex, but not the NSAID carp, increased trabecular bone density in sham-operated female mice

**Figure 1:**
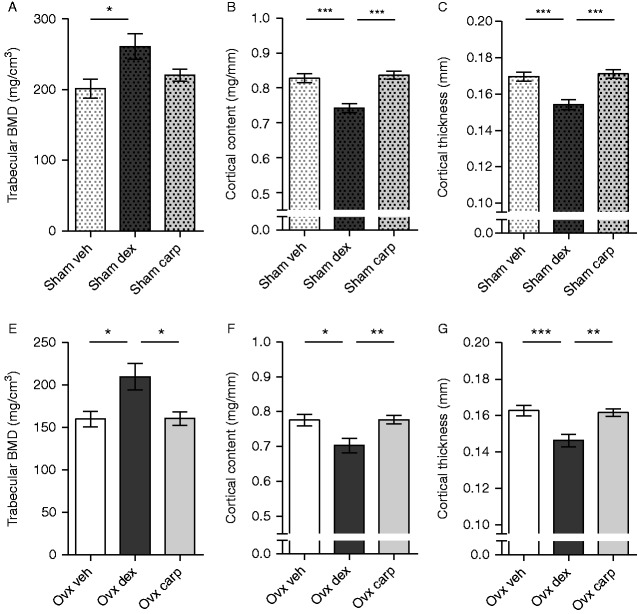
Dex, but not carp, increased trabecular bone density in sham-operated and ovx mice. (A) Trabecular BMD, (B) cortical content, and (C) cortical thickness analyzed by pQCT in femurs from sham-operated C57BL/6 mice treated with 125 μg/mouse of dex (*n*=13), 125 μg/mouse of carp (*n*=13), or vehicle (veh, *n*=12) and (D) trabecular BMD, (E) cortical content, and (F) cortical thickness in femurs from ovx C57BL/6 mice treated with 125 μg/mouse of dex (*n*=12), 125 μg/mouse of carp (*n*=13), or vehicle (veh, *n*=12). Data are the arithmetical mean±s.e.m. *P<0.05, **P<0.01, and ***P<0.001. ANOVA, Tukey's *post hoc* test.

Sham-operated female C57BL/6 mice were treated for 2.5 weeks with dex (125 μg/day) or vehicle control to investigate how dex affects bone. To compare the effect of dex with another anti-inflammatory drug, a group of mice treated with the NSAID carp (125 μg/mouse) were also included in the study. Dex-treated mice showed a 30% increase in trabecular BMD compared with vehicle-treated mice (Fig. 1A). However, the cortical content and cortical thickness were decreased in dex-treated compared with vehicle-treated mice (Fig. 1B and C). In contrast to dex-treated animals carp-treated mice did not differ compared with vehicle-treated mice with respect to trabecular BMD, cortical content, or cortical thickness (Fig. 1).

### Treatment with dex, but not carp, increased trabecular bone in ovx mice

To determine how dex alters bone density in ovx mice, a model of postmenopausal osteoporosis, C57BL/6 mice were treated for 2.5 weeks with dex, carp, or vehicle control. In line with the results from sham-operated mice (Fig. 1A), dex-treated ovx mice showed a 31% increase in trabecular BMD compared with vehicle-treated mice (Fig. 1D). In addition, the cortical content and cortical thickness were decreased in dex-treated compared with vehicle-treated ovx mice (Fig. 1E and F). Carp-treated mice did not differ from vehicle-treated mice with respect to trabecular BMD, cortical content, or cortical thickness (Fig. 1). In addition, there was an effect of ovx on trabecular BMD (*P*=0.02, Student's *t*-test) and cortical content (*P*=0.02, Student's *t*-test), but not on cortical thickness (*P*=0.08, Student's *t*-test), when comparing vehicle-treated ovx and sham mice (Fig. 1).

**Figure 2:**
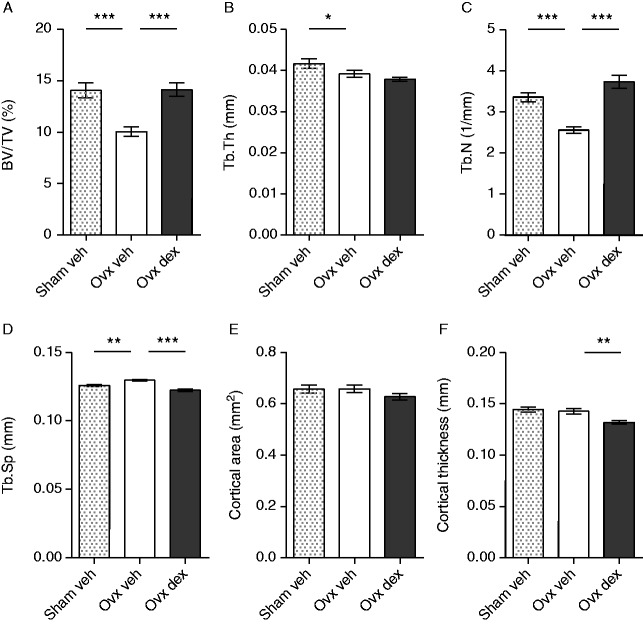
Dex increased trabecular bone volume described as a percentage of tissue volume and trabecular number in ovx mice. (A) Trabecular bone volume as a percentage of tissue volume (TB/TV), (B) trabecular thickness (Tb.Th), (C) trabecular number (Tb.N), (D) trabecular separation (Tb.Sp), (E) cortical area, and (F) cortical thickness analyzed by μCT in femurs from sham-operated C57BL/6 mice treated with vehicle (veh, *n*=10) or ovx C57BL/6 mice treated with 125 μg/mouse of dex (*n*=10) or vehicle (veh, *n*=10). Data are the arithmetical mean±s.e.m. *P<0.05, **P<0.01 and ***P<0.001. ANOVA, Tukey's (A, C, D, E, and F) or Dunnett's (B) *post hoc* test.

**Figure 3:**
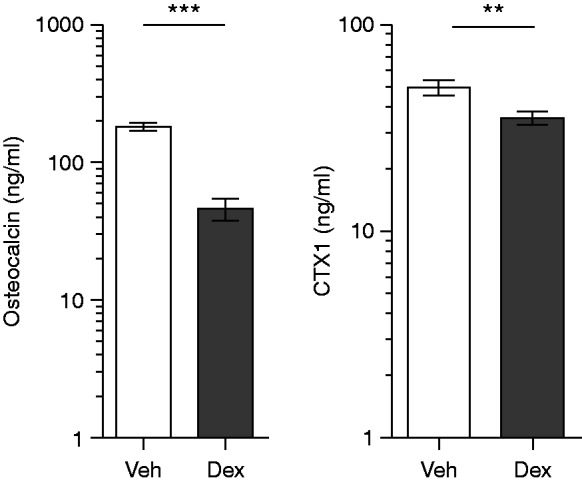
Dex decreased markers of bone turnover in serum of ovx mice. (A) The bone formation marker osteocalcin and (B) the bone resorption marker CTX1 were determined in serum from ovx C57BL/6 mice treated with 125 μg/mouse of dex (*n*=10) or vehicle (veh, *n*=10). **P<0.01, and ***P<0.001. Data are the geometric mean±95% CI. Student's *t*-test.

To more carefully dissect the effect of dex on bone, μCT analyses were performed in a separate experiment. As expected, ovx induced a decrease in BV/TV, trabecular thickness (Tb.Th), and trabecular number (Tb.N) when comparing vehicle-treated ovx and sham mice, while the trabecular separation (Tb.Sp) was increased (Fig. 2A, B, C, and D). Ovx had no effect on cortical parameters (Fig. 2E and F). BV/TV and Tb.N was increased in dex-treated compared with vehicle-treated ovx mice, while the Tb.Sp was decreased, and the Tb.Th was unaltered (Fig. 2A, B, C, and D). The cortical area did not differ between groups (Fig. 2E), but the cortical thickness measured by μCT was decreased in dex-treated compared with vehicle-treated ovx mice (Fig. 2F). In these mice, bone formation and resorption were assessed by serum analysis of osteocalcin and CTX1, and dex clearly decreased levels of the two markers in ovx mice, by 81 and 28% respectively (Fig. 3).

### Dex dose-dependently affects bone density

**Figure 4:**
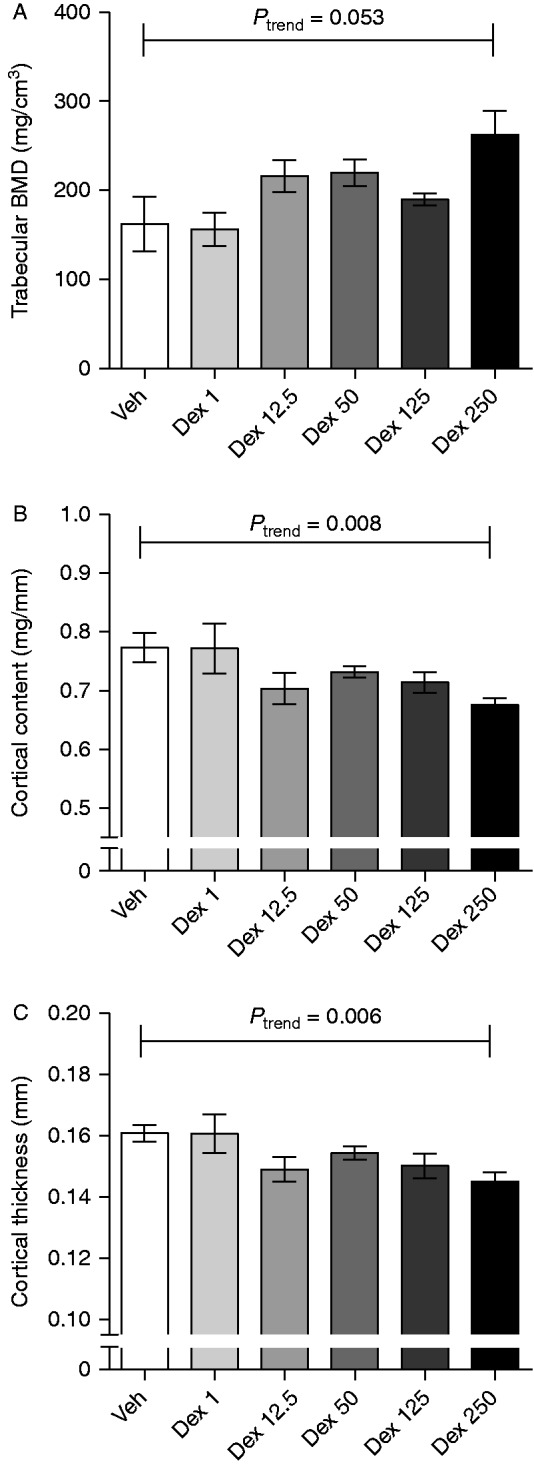
Dex dose-dependently affected bone parameters measured by pQCT in ovx mice. (A) Trabecular BMD (β=0.243), (B) cortical content (β=−3×10^4^), and (C) cortical thickness (β=−5×10^4^) analyzed by pQCT in femurs from ovx C57BL/6 mice treated with 1 (*n*=6), 12.5 (*n*=6), 50 (*n*=6), 125 (*n*=7), or 250 (*n*=6) μg/mouse of dex or vehicle (veh, *n*=6). Data are the arithmetical mean±s.e.m. Linear regression was used to test for a dose-dependent trend.

**Figure 5:**
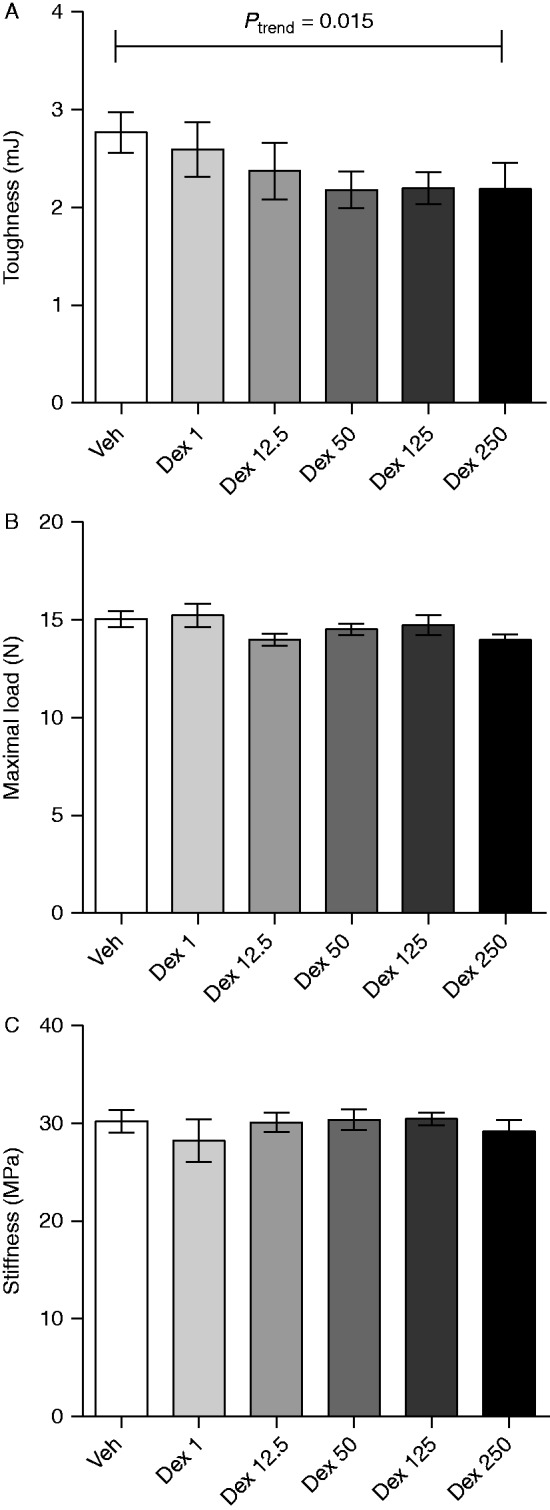
Dex dose-dependently decreased the toughness, but not the stiffness or load resisted by the bone in ovx mice. (A) Toughness (energy absorption at maximal load, β=−0.003), (B) maximal load at failure (β=−0.003), and (C) stiffness (MPa, β=−0.0004) analyzed by three-point bending test in tibiae from ovx C57BL/6 mice treated with 1 (*n*=6), 12.5 (*n*=6), 50 (*n*=6), 125 (*n*=7), or 250 (*n*=6) μg/mouse of dex or vehicle (veh, *n*=6). Data are the arithmetical mean±s.e.m. Linear regression was used to test for a dose-dependent trend.

To determine whether the bone effects of dex were dose-dependent, ovx mice were treated with 1, 12.5, 50, 125, or 250 μg/mouse per day of dex. Cortical content and thickness were dose dependently decreased by dex, and trabecular BMD seemed to be dose-dependently increased by dex, although not significantly (Fig. 4). As cortical content and cortical thickness were decreased in dex-treated mice, we wanted to investigate whether the bone strength was also affected by subjecting the tibia to a three-point bending test. Dex dose-dependently decreased the toughness (Fig. 5A), but not the maximal load resisted by the bone or the stiffness (Fig. 5B and C).

### Dex may exert its bone remodeling effects via lymphocytes

**Figure 6:**
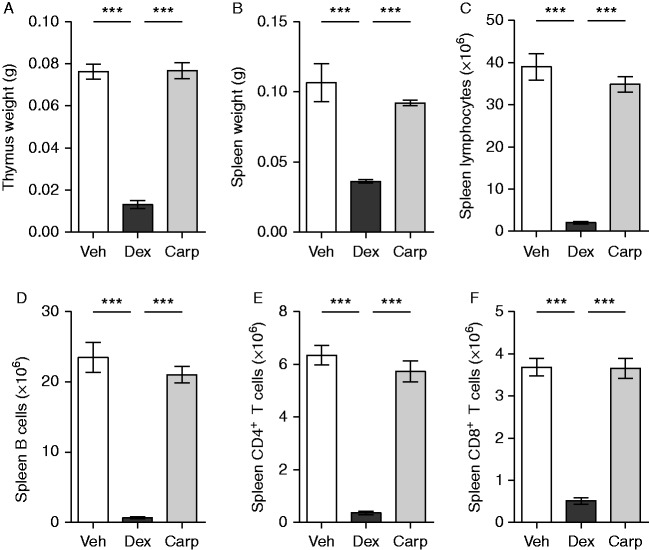
Dex decreased thymus and spleen weight, and the number of spleen lymphocytes in ovx mice. (A) Thymus weight and (B) spleen weight were measured in ovx C57BL/6 mice treated with 125 μg/mouse of dex (*n*=12), 125 μg/mouse of carp (*n*=13), or vehicle (Veh, *n*=13). In these mice, the numbers of spleen (C) lymphocytes, (D) B cells, (E) CD4^+^ T cells, and (F) CD8^+^ T cells were analyzed by flow cytometry. Data are the arithmetical mean±s.e.m. ***P<0.001. ANOVA, Tukey's (A) or Dunnett's T3 (B, C, D, E, and F) *post hoc* test.

**Figure 7:**
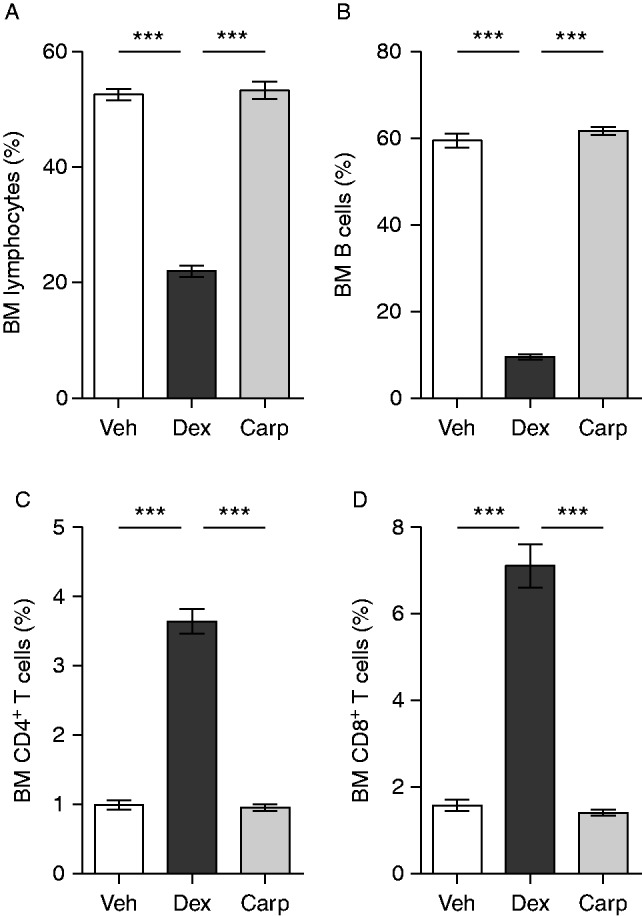
Dex decreased the proportion of bone marrow (BM) lymphocytes in ovx mice. The percentage of (A) lymphocytes (percentage of singlets), (B) B cells (percentage of lymphocytes), (C) CD4^+^T-cells (percentage of lymphocytes), and (D) CD8^+^ T cells (percentage of lymphocytes) were analyzed by flow cytometry in bone marrow from ovx C57BL/6 mice treated with 125 μg/mouse of dex (*n*=9), 125 μg/mouse of carp (*n*=11), or vehicle (veh, *n*=10). Data are the arithmetical mean±s.e.m. ***P<0.001. ANOVA, Tukey's (A) or Dunnett's T3 (B, C, and D) *post hoc* test.

Both dex and carp are anti-inflammatory, but the mechanisms differ, for example, in that dex has lymphocyte-suppressive effects (Fauci 1976, Tuckermann *et al*. 2005). Indeed, treatment with dex decreased the thymus and spleen weights compared with vehicle- and carp-treated ovx mice (Fig. 6A and B). The numbers of total lymphocytes, B cells, CD4^+^ T cells, and CD8^+^ T cells in spleen were severely decreased in dex-, but not in carp-treated ovx mice, when compared with vehicle-treated mice (Fig. 6C, D, E and F). In bone marrow, dex also decreased the frequencies and number of total lymphocytes and B cells in ovx mice, but increased the frequencies and numbers of CD4^+^ T cells and CD8^+^ T cells compared with vehicle and carp (Fig. 7 and Table 1). In sham-operated mice treated with dex and carp, all results regarding organ weights and cell populations were found to be the same as those for ovx mice (data not shown).

**Figure 8:**
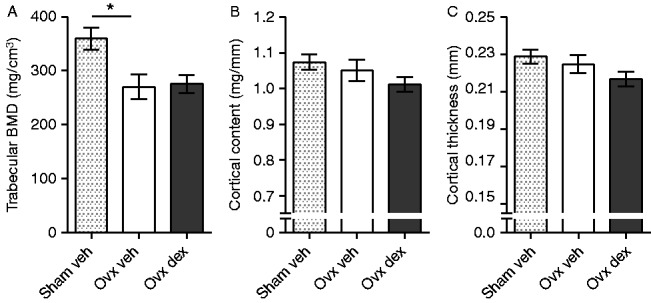
Dex did not increase trabecular bone density in ovx SCID mice. (A) Trabecular BMD, (B) cortical content, and (C) cortical thickness analyzed by pQCT in femurs from sham-operated SCID mice treated with vehicle (veh, *n*=11), and ovx SCID mice treated with 125 μg/mouse of dex (*n*=12) or vehicle (veh, *n*=11 in A, *n*=10 in B and C). Data are the arithmetical mean±s.e.m. *P<0.05. ANOVA, Dunnett's T3 (A) or Tukey's (B and C) *post hoc* test.

As we found that dex, but not carp, affected lymphocyte numbers, we proposed the hypothesis that the bone remodeling effects of dex are exerted via lymphocytes. SCID mice that lack lymphocytes were therefore ovariectomized and treated with dex or vehicle. Indeed, the dex-treated ovx SCID mice did not differ from vehicle-treated ovx mice regarding trabecular BMD, cortical content, and cortical thickness (Fig. 8). However, similarly to C57BL/6 mice, SCID mice displayed an ovx-induced decrease in trabecular BMD compared with vehicle-treated sham mice (Fig. 8A).

## Discussion

The results of this study indicate that treatment with the glucocorticoid dex had beneficial effects on trabecular bone as it increased trabecular BMD in sham-operated female C57BL/6 mice. This effect was also observed in ovx mice, a model of postmenopausal osteoporosis. In these mice, dex also increased the trabecular bone volume as percentage of tissue volume, and Tb.N, while it decreased the Tb.Sp. In spleen, the numbers of lymphocytes, both T and B cells, were greatly diminished in dex-treated mice. These findings were not seen when mice were treated with the NSAID carp, possibly indicating that the mechanism is specific to dex, rather than a general anti-inflammatory effect. To investigate the role of lymphocytes on the dex-mediated effects on bone, SCID mice lacking lymphocytes were ovariectomized and treated with dex. Dex treatment had no effect on BMD in these mice.

Cortisol and estrogen can interact at the receptor level (Miranda *et al*. 2013), but dex increased trabecular bone in both sham-operated and ovx mice, which implies that this finding is independent of estrogen status. This suggestion is further strengthened by the finding that the cortical content and thickness were decreased in both groups of mice. Dex treatment clearly results in a redistribution of bone from the peripheral (cortical) to the central (trabecular) bone compartment. The reason for this is not fully understood. However, it is interesting to note that glucocorticoids redistribute fat from subcutaneous depots in the limbs to those in the central regions of the body, i.e. the intra-abdominal depot and face (Mayo-Smith *et al*. 1989).

A possible mechanism for the bone remodeling effects of dex is via its well-known actions on lymphocytes (Fauci 1976, Tuckermann *et al*. 2005). Lymphocytes have been shown to play a negative role in ovx- and arthritis-induced osteoporosis by producing pro-inflammatory cytokines and RANKL, thereby inducing osteoclasts and inhibiting osteoblasts (Cenci *et al*. 2000, Pacifici 2008, Islander *et al*. 2011*b*). The role of lymphocytes in homeostasis in healthy bone is less well studied. However, results from one study were indicative of a beneficial role, as lymphocytes stimulated the production of the anti-osteoclastogenic factor osteoprotegerin (Li *et al*. 2007). In contrast, results from another study indicated that inhibition of T cells, through administration of CTLA-4Ig, increased bone mass (Roser-Page *et al*. 2014). The results of this study indicate that dex treatment greatly reduced spleen and bone marrow lymphocytes, while the NSAID carp had no effect on lymphocytes or bone. Furthermore, in ovariectomized SCID mice that lack lymphocytes, dex treatment did not result in increased trabecular BMD. Together, these results indicate that the mechanism for dex's protective role on trabecular bone may indeed be mediated via its effect on lymphocytes. However, further studies are needed to verify this hypothesis.

Although glucocorticoids have suppressive effects on lymphocytes, the magnitude varies between locations and subsets of lymphocytes. Glucocorticoids dramatically reduce immature thymic T cells and bone marrow B cell precursors through apoptosis and also induce circulating lymphocytopenia in T cells in particular (Fauci 1975, Igarashi *et al*. 2005, Thompson 2008, Dimitrov *et al*. 2009). However, glucocorticoids have been shown to increase T and B cells in the bone marrow. This may be due to redistribution from the circulation, rather than apoptosis, as the effect is transient (Fauci 1975, Dimitrov *et al*. 2009). For T cells, glucocorticoids mainly deplete naïve and central memory T cells from the circulation, possibly this is dependent on their high expression of the bone marrow homing receptor CXCR4 (Dimitrov *et al*. 2009). Consistent line with this, we found an increase in T cells, but a large decrease in B cells in the bone marrow. The latter may be due to an increased tendency of T cells to redistribute to the bone marrow compared with B cells (Fauci 1975). Another possible explanation may be a higher tendency towards apoptosis in B cells, as they were greatly decreased in both bone marrow and spleen of dex-treated mice. However, the exact mechanism causing the lymphocyte alterations needs to be further investigated. To verify the hypothesis that dex improves trabecular bone through its effect on lymphocytes, and to specify the cell subset of interest, it would be of importance to transplant T or B cells from wild-type mice to SCID mice lacking lymphocytes, and then treat these animals with dex. Another suggestion for future work in order to verify our hypothesis would be to cell-specifically knock out the glucocorticoid receptor in T and/or B cells.

The finding that the glucocorticoid dex displayed beneficial effects on trabecular bone is somewhat controversial as studies carried out using several different species have shown adverse effects on bone (Ferretti *et al*. 1995, Weiler *et al*. 1995, Van Staa *et al*. 2000b, Takahashi *et al*. 2006). In addition, glucocorticoid treatment in combination with ovx has displayed adverse effects on bone in rats (Govindarajan *et al*. 2013). However, besides the findings that glucocorticoid-treated arthritic mice and human rheumatoid arthritis patients maintained their BMD (Svensson *et al*. 2005, Islander *et al*. 2011*a*), there are studies showing improvements in trabecular and/or total bone in intact male rats treated with glucocorticoids (Binz *et al*. 1994, King *et al*. 1996, Ogoshi *et al*. 2008). There is also a study on young rabbits showing increased trabecular BMD and decreased cortical bone after glucocorticoid treatment (Kugelberg *et al*. 2005). Many postmenopausal women are treated with glucocorticoids for various diseases, and they have been found to have more asymptomatic vertebral fractures than healthy postmenopausal women (Angeli *et al*. 2006). However, the underlying disease may interfere with the results, and the data on healthy women in the study mentioned were taken from epidemiological studies rather than being included as proper controls. In contrast, a retrospective study on postmenopausal women with lower back pain did not find a correlation between fractures or BMD and the dose or duration of glucocorticoid treatment (Kwok *et al*. 2012).

Glucocorticoids have previously been shown to decrease bone formation through direct effects on bone cells (Weinstein *et al*. 1998). Indeed, the bone resorption and formation markers CTX1 and osteocalcin were greatly decreased in dex-treated ovx mice, indicating low bone turnover. However, these results do not explain the increase in trabecular and decrease in cortical bone found in this study. A possible explanation for the discrepancy between bone turnover markers and bone morphology is that CTX1 and osteocalcin reflect bone turnover both in trabecular and cortical bone. It is therefore difficult to draw conclusions regarding the turnover in a certain compartment. Also, osteocalcin may not only indicate bone formation but also bone resorption (Delmas 1993). At least in humans, the currently recommended marker of bone formation is serum procollagen type 1 N propeptide (Vasikaran *et al*. 2011).

A limitation with this study is the use of serum markers as a measure of bone turnover, instead of histomorphometry. Another limitation is the lack of data on trabecular bone strength, as the three-point bending test used in this study mainly reflects cortical strength. In this study, dex does not have any large effect on the different measurements of bone strength. The reason for this may be the rather short duration of treatment. Indeed, the dose-dependency study showed a relatively low effect of dex on cortical content, in contrast to the more quickly responding trabecular bone. As cortical content and mechanical parameters are highly correlated, a large effect on the latter is thus not likely (Jamsa *et al*. 1998). In addition to the three-point bending test, it would have been interesting to perform a vertebral compression test or femur cantilever test, which reflect trabecular bone properties to a larger extent. Also, SCID mice are on a C.B-17 background, and the lack of dex-induced bone effects in these mice might thus reflect differences in background strain compared with C57BL/6 mice. Nevertheless, this resistance to glucocorticoid treatment is not generalized as both SCID and/or C.B-17 mice display reduced spleen weight and cellularity, thymus weight and cellularity, and bone marrow cellularity in response to dex (C Jochems, U Islander and H Carlsten unpublished observations). Moreover, C57BL/6 and SCID mice are similar in that they both show an ovx-induced bone loss. However, to fully avoid any possible effects of background differences, C57BL/6 mice lacking T and B cells, such as *Rag1* or *Rag2* knockouts, could be interesting to investigate in future studies.

In conclusion, the results of this study indicate that dex increases trabecular BMD through its lymphocyte-altering effects. This adds to the body of evidence that glucocorticoids do not only have detrimental effects on bone. Similar effects on bone were found in sham-operated and ovx mice, which implies that the dex-induced gain in trabecular bone is independent of estrogen status. However, the findings in ovx mice, as a model of postmenopausal osteoporosis, are more relevant to the clinical situation. An appealing option would be to find the exact mechanism for the protective effect of dex on trabecular bone in order to tailor a drug that isolates this effect. That would allow the drug to bypass the adverse effects of dex on cortical bone.

## Author contribution statement

L G, C J, C O, U I, and H C designed the experiments; L G, C J, A A, C E, and U I performed the experiments; L G, C J, A A, and U I analyzed the data; C O and H C contributed reagents/materials/analysis tools; L G, C J, A A, C E, C O, U I, and H C prepared and approved the final version of the manuscript.

## Figures and Tables

**Table 1:** Number of bone marrow lymphocytes analyzed by flow cytometry from ovx C57BL/6 mice treated with 125 μg/mouse of dex, 125 μg/mouse of carp, or vehicle. Data are the arithmetical mean±s.e.m. ANOVA with Tukey's *post hoc* test was used for lymphocyte numbers, CD4^+^ and CD8^+^ T cells, and Dunnetts's T3 *post hoc* test for B cell numbers

	**Lymphocytes** (×10^6^)	**B cells** (×10^6^)	**CD4^+^ T cells** (×10^6^)	**CD8^+^ T cells** (×10^6^)
Ovx veh	22.8±0.8	13.6±0.7	0.23±0.01	0.36±0.03
Ovx dex	14.4±0.8^*^	1.4±0.1^*^	0.53±0.04^*^	1.04±0.10^*^
Ovx carp	23.0±0.9^†^	14.1±0.5^†^	0.22±0.02^†^	0.33±0.02^†^

*P<0.001, ovx dex versus ovx veh and ^†^*P*<0.001, ovx carp versus ovx veh.
